# The EIF4E1-4EIP cap-binding complex of *Trypanosoma brucei* interacts with the terminal uridylyl transferase TUT3

**DOI:** 10.1371/journal.pone.0258903

**Published:** 2021-11-22

**Authors:** Franziska Falk, Kevin Kamanyi Marucha, Christine Clayton

**Affiliations:** DKFZ-ZMBH Alliance, Center for Molecular Biology of Heidelberg University (ZMBH), Heidelberg, Germany; John Curtin School of Medical Research, AUSTRALIA

## Abstract

Most transcription in *Trypanosoma brucei* is constitutive and polycistronic. Consequently, the parasite relies on post-transcriptional mechanisms, especially affecting translation initiation and mRNA decay, to control gene expression both at steady-state and for adaptation to different environments. The parasite has six isoforms of the cap-binding protein EIF4E as well as five EIF4Gs. EIF4E1 does not bind to any EIF4G, instead being associated with a 4E-binding protein, 4EIP. 4EIP represses translation and reduces the stability of a reporter mRNA when artificially tethered to the 3’-UTR, whether or not EIF4E1 is present. 4EIP is essential during the transition from the mammalian bloodstream form to the procyclic form that lives in the Tsetse vector. In contrast, EIF4E1 is dispensable during differentiation, but is required for establishment of growing procyclic forms. In *Leishmania*, there is some evidence that EIF4E1 might be active in translation initiation, via direct recruitment of EIF3. However in *T*. *brucei*, EIF4E1 showed no detectable association with other translation initiation factors, even in the complete absence of 4EIP. There was some evidence for interactions with NOT complex components, but if these occur they must be weak and transient. We found that EIF4E1is less abundant in the absence of 4EIP, and RNA pull-down results suggested this might occur through co-translational complex assembly. We also report that 4EIP directly recruits the cytosolic terminal uridylyl transferase TUT3 to EIF4E1/4EIP complexes. There was, however, no evidence that TUT3 is essential for 4EIP function.

## Introduction

The rates at which translation initiation and elongation occur determine the amount of protein that is synthesized from a particular mRNA [1]. Cap-binding proteins exert essential roles during the initiation process. Classically, the cap is bound by an eIF4E protein, which in turn recruits an eIF4G protein. eIF4G serves as a scaffold for further association with the helicase eIF4A and the 43S complex, which consists of the small ribosomal subunit and several initiation factors. Once the latter has identified the first start codon through scanning, the large ribosomal subunit is recruited, allowing translation to proceed [2]. Most eukaryotic species studied possess more than one eIF4E homolog, the activities of which can either be restricted to a certain subset of mRNAs, or affect bulk translation [3].

Translation can be regulated through several mechanisms, many of which act upon the initiation process. In animal cells, binding of 4E-binding proteins (4E-BPs) and eIF4G proteins to eIF4Es is mutually exclusive, with the 4E-BP/eIF4E interaction leading to a blockade of productive initiation complex assembly [4]. Binding of 4E-BPs to eIF4Es is mediated through a YXXXXLØ motif near the 4E-BP N-terminus, which is shared with eIF4Gs [5]. In contrast, eIF4E-like cap-binding proteins of the 4EHP-type do not bind to eIF4G proteins and have suppressive functions. They are exemplified by 4EHP/eIF4E2, d4EHP/EIF4E-8, and nCBP in mammals, *Drosophila*, and *Arabidopsis*, respectively [6–8]. Their suppressive functions appear to be mediated through their association with partner proteins, such as Bicoid or GIGYF2, which independently display RNA-binding activities [9–13]. Association of mRNAs with either 4E-BP or 4EHP and their binding partners results in the recruitment of degradation machineries, such as deadenylation complexes, leading to reduced mRNA stability as well as reduced translation [12, 14]. The 4EHP/GIGYF2 complex was recently implicated in co-translational destruction of mRNAs after ribosome pausing [15–17].

*Trypanosoma brucei* are unicellular eukaryotic parasites, members of the order Kinetoplastida which also includes *Leishmania* species. Kinetoplastids regulate gene expression primarily through post-transcriptional mechanisms, as transcription by RNA polymerase II is constitutive and polycistronic [18]. However, they are faced with a high demand for plasticity in terms of gene expression profiles, as nutrient sources, ambient temperature, host defense mechanisms, and other factors differ vastly between the hosts encountered during the life cycle. For *Trypanosoma brucei* in the bloodstream of a mammalian host, glucose serves as the main source for ATP production, and evasion of the immune response by antigenic variation of surface proteins is required for survival [19–21]. As trypanosome cell densities reach a critical level, development of long slender bloodstream forms into stumpy forms is induced. Growth arrest and low overall mRNA levels and translation rates are hallmark features of the stumpy stage, but expression of a selected set of proteins is strongly activated, including the surface protein PAD1 and some proteins of mitochondrial energy metabolism [22–24]. The stumpy form is primed for differentiation into procyclic forms upon uptake by a tsetse fly through a blood meal. Procyclic forms express EP and GPEET procyclins on their surface, and amino acids serve as the main sources for energy production [25, 26].

In accordance with their need for adaptive translation regulation, kinetoplastid parasites are equipped with six eIF4E isoforms (EIF4E1-6), as well as five eIF4Gs (EIF4G1-5), the individual roles of which have been the subject of numerous studies [27]. EIF4Es 3–6 have EIF4G partners and are probably all active in translation initiation, while the role of EIF4E2 is unclear [28, 29]. Much of the evidence concerning EIF4E1 comes from *Leishmania*, which grow intracellularly as amastigotes within vertebrates, and as promastigotes within the sandfly vector. *Leishmania* EIF4E1 does not interact with any of the EIF4Gs, instead associating with a protein called 4E-interacting protein, 4EIP, which has the canonical EIF4E binding motif YXXXXLØ at its N-terminus [30]. A second EIF4E-binding protein, 4EBP2, was also recently found to associate with either EIF4E1 or EIF4E3 [31]. *Leishmania* promastigotes that completely lacked EIF4E1 showed altered morphology, with impaired growth and reduced overall translation and metabolism; the proteome changes in the mutant cells reflected their greatly reduced flagellar length. The ability of these cells to infect, and multiply within, macrophages was reduced, but not completely eliminated [32].

After *Leishmania* EIF4E1 purification, some general translation initiation factors, including EIF3 and EIF2 subunits, were detected. In addition, tagged *Leishmania* EIF3A is able to pull down EIF4E1 *in vitro*, and a yeast 2-hybrid interaction between EIF3A and EIF4E1 was demonstrated [33], suggesting that *Leishmania* EIF4E1 might act in translation initiation by direct EIF3 recruitment. Structural and *in vitro* studies characterised *Leishmania* EIF4E1 binding to an N-terminal fragment of 4EIP, and showed that 4EIP inhibits EIF4E1 binding to a cap analogue [34]. These results would all be consistent with a model in which *Leishmania* EIF4E1 is an EIF4G-independent translation initiation factor whose activity is inhibited by 4EIP and perhaps also by 4EIP2.

Results with *T*. *brucei* have confirmed the interaction between EIF4E1 (Tb927.11.2260) and 4EIP (Tb927.9.11050), but have also suggested that EIF4E1 and 4EIP might have some independent activities. When tethered via a λN peptide to a boxB-bearing reporter mRNA, *T*. *brucei* 4EIP strongly represses reporter expression whether or not EIF4E1 is present [35, 36]. This could mean that the tethering is merely replacing EIF4E1 in recruiting 4EIP to RNA, since similar activities have also been shown for metazoan 4E-BPs [14, 37]. However we showed that 4EIP, like GIGYF2, is directly cross-linkable to mRNAs [38], which might allow it to function independently.

Although bloodstream forms lacking either EIF4E1 or 4EIP have only a mild growth defect, 4EIP is essential for full differentiation to the stumpy form; without 4EIP, the translational repression that is intrinsic to this process is delayed and the cells are no longer able to differentiate fully to procyclic forms [35]. Notably, a truncated form of 4EIP that cannot bind to EIF4E1 can restore stumpy formation in the 4EIP-deficient cells [35]. Meanwhile, initial differentiation of EIF4E1-deficient cells proceeds normally. These observations suggest a function of 4EIP that is independent of EIF4E1. Although tethering of EIF4E1 causes repression when 4EIP is present, in the absence of 4EIP tethering of EIF4E1 has no effect at all. One observation, however, suggests that EIF4E1 has an independent function; unlike 4EIP, we were unable to obtain procyclic trypanosomes lacking EIF4E1 [35]. Apart from this, Mabille et al. highlighted that 4EIP is essential for *in vivo* infectivity in the vertebrate host, as evidenced by a failure to detect 4EIP KO parasites after one week of infection [39]; immunosuppressive treatment could not rescue the defect.

This paper also concerns a *T*. *brucei* terminal uridylyl transferase. In mammalian cells and *Schizosaccharomyces pombe*, addition of uridine residues to the 3’-ends of RNAs makes them substrates of the 3’-5’ exoribonuclease Dis3L2 [40], with subsequent degradation by the exosome. Substrates include U6 snRNA in the nucleus, and miRNAs and the non-polyadenylated histone mRNAs in the cytosol [41, 42]. *T*. *brucei* has five proteins with strong similarity to terminal uridylyl transferases [43]. Three of them are implicated in mitochondrial RNA editing [44]. One of the remaining ones, TUT3 (Tb927.10.7310), is a highly processive terminal uridylyl transferase, lacks a mitochondrial targeting signal and, by cell fractionation, was not in the mitochondria of *Leishmania tarentolae* [43]. Neither the level of the *TUT3* mRNA, nor its translation, are developmentally regulated in *T*. *brucei* [45] and mass spectrometry results suggest that it has low abundance and is absent from mitochondria [46, 47]. A Dis3L2 homolog (Tb927.11.8290) is present, but the protein is concentrated in the nucleus [48]. In a high-throughput RNAi screen using the Lister 427 strain of *T*. *brucei*, which is unable to make stumpy forms or transform to growing procyclic forms, RNAi targeting either TUT3 or the Dis3L2 homologue had no effect on bloodstream form or procyclic form trypanosome survival [49].

The main aim of the current study was to clarify the possible roles of EIF4E1 both with, and independent of 4EIP. We found no evidence that EIF4E1 can act as a translation initiation factor, but did discover an unexpected interaction between 4EIP and TUT3.

## Results

### The abundance of EIF4E1 decreases in the absence of 4EIP

**Fig 1:**
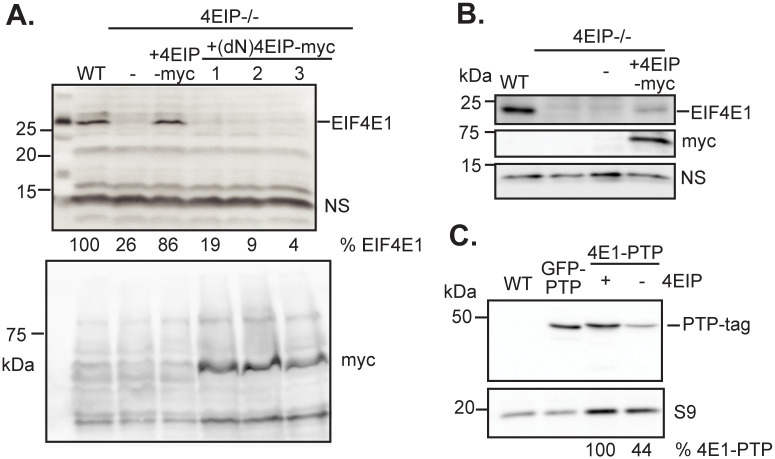
EIF4E1 abundance is reduced in the absence of 4EIP. (A) Expression of EIF4E1 was analysed in WT and 4EIP-deficient procyclic forms, as well as in 4EIP-deficient cells reconstituted with myc-tagged full-length or N-terminally deleted 4EIP. NS: non-specific band used as a control; S9: 40S ribosomal protein S9. (B) Expression of EIF4E1 was analysed by western blotting in WT, EIF4E1- and 4EIP-deficient bloodstream forms, as well as in 4EIP-deficient cells reconstituted with full-length, myc-tagged 4EIP. (C) Western blot analysis of PTP-tagged versions of EIF4E1 (± 4EIP) and GFP, which were introduced into pleomorphic bloodstream forms of *T*. *brucei*. These could be switched to procyclic forms by *cis*-aconitate treatment (not shown) and were used for mass spectrometry.

T. brucei EIF4E1 is present at 3–8 x10^3^ molecules/cell in procyclic forms, and 1.5–5 x10^3^ molecules/cell in bloodstream forms, as judged by quantitative Western blotting with protein from the Lister 427 strain [50]. Quantitative proteomic results suggested roughly 1.5-fold increased expression in procyclic forms [46]. All experiments in this paper were done with differentiation-competent EATRO1125 forms. Western blot results (S1A Fig) confirmed that the protein was detectable in both forms, but specific bands were quite weak. Intriguingly, the abundance of EIF4E appeared to be reduced in the absence of 4EIP (Fig 1A and 1B). In procyclic forms, expression of EIF4E1 was rescued upon ectopic expression of full-length 4EIP-myc (Fig 1A). (Note that full-length 4EIP-myc was difficult to detect, usually we saw only a degraded version.) A deletion mutant of 4EIP lacking the N-terminal interaction motif for binding to EIF4E1 was expressed well, but unable to rescue EIF4E1 expression (Fig 1A), suggesting that complex formation was required to maintain EIF4E1 abundance. EIF4E1 abundance was also reduced in bloodstream forms lacking 4EIP, but rescue through expression of 4EIP was incomplete (Fig 1B, S1B Fig); we do not know why.

To follow EIF4E1 expression more easily in wild-type (WT) and 4EIP knockout (KO) backgrounds, as well as for subsequent studies, we introduced a sequence encoding a PTP-tag into the genomic *EIF4E1* locus in bloodstream forms, joining the tag to the protein C-terminus (Fig 1C). These cells were them differentiated to procyclic forms, where deletion of the second gene copy gave no growth defect (division time of about 9.5 h, similar to wild-type), confirming the function of the fusion protein. EIF4E1-PTP was also expressed in cells lacking 4EIP; this was done in separate transfections since the deletion mutant cannot differentiate. The amounts of PTP-tagged EIF4E1 were also lower in the 4EIP-null cells (Fig 1C). These results together suggest that interaction of 4EIP with EIF4E1 is required in order to maintain normal levels of EIF4E1.

*In situ* C-terminal tagging replaces the mRNA 3’-untranslated region, which is normally important for expression regulation; it is therefore possible that abnormal levels of total EIF4E1 are produced in the tagged cells. However, the requirement for 4EIP to maintain the protein is likely to counteract such effects.

### Association with general translation initiation factors, the NOT complex and TUT3

It was previously suggested that *Leishmania* EIF4E1 acts as a translation factor which is inhibited by 4EIP [33]. One piece of evidence was the *in vitro* and two-hybrid interaction between *Leishmania* EIF4E1 and EIF3A [33].

**Fig 2:**
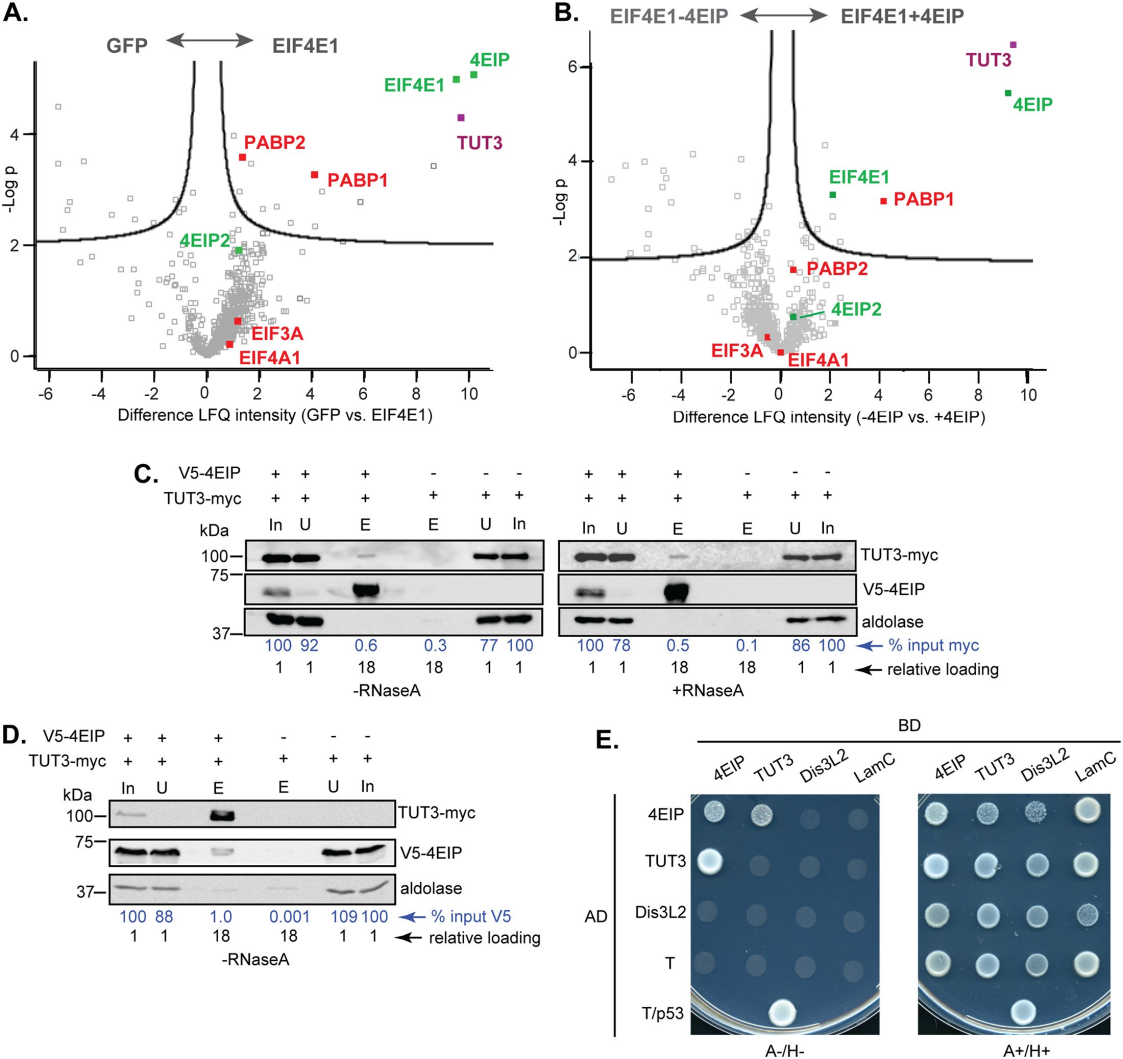
Protein binding partners of EIF4E1 in *Trypanosoma brucei* procyclic forms. **(A)** PTP-tagged GFP and EIF4E1 were pulled down from procyclic forms for comparison of bound proteins using quantitative mass spectrometry. The image was produced using Perseus [63] and shows the log_2_ relative enrichment on the x-axis and the negative adjusted log_10_ P-value on the y-axis. Each square represents one protein and some proteins of interest are highlighted in colour. Proteins with high enrichment (towards the right) and low P-values (high -logP) were enriched in the EIF4E1 preparation. In this analysis, the abundances of proteins that were completely absent in the GFP-PTP preparation were simulated by the programme. Note that there was no significant enrichment of EIF4A1, the translation-linked RNA helicase, or EIF3A. **(B)** PTP-tagged EIF4E1 was pulled down from procyclic forms that either had 2 copies of the 4EIP gene (+4EIP, same dataset as in A), or no copies (-4EIP). For details of the plot see (A). Proteins that are associated either in the presence or absence of 4EIP, such as 4E-IP2, now show almost no relative enrichment. EIF4E1 is more enriched in the presence of 4EIP because it is more abundant in those cells. **(C)** Pull-downs of V5-tagged 4EIP from bloodstream form *Trypanosoma brucei* parasites in absence or presence of RNase A (left and right panels, respectively), followed by detection of TUT3-myc enrichment by western blotting. In each pull-down, 5% each of the input (In) and unbound (U) fractions were loaded as controls. The eluate from the beads (E) includes the remainder of the sample. The densitometry shows the relative amounts of TUT3-myc in input, wash and eluates, adjusted for the loading. **(D)** Pull-down of myc-tagged TUT3 from bloodstream-form *T*. *brucei* parasites, followed by detection of 4EIP-V5 by western blotting. Other details are as in (C) except that now the relative amounts of V5-4EIP are shown. **(E)** Yeast-2-hybrid assays for detection of direct interactions between 4EIP, TUT3, and the exonuclease DIS3L2. Proteins that interact give growth on plates lacking adenine and histidine (A-/H-). The panel on the right (A+/H+) is a plate containing adenine and histidine, but with selection for both plasmids; this shows that in all cases, the cells contained both plasmids. The interaction between large T antigen (T) and p53 served as positive control. The combination of expression vectors encoding lamin C (LamC) and T served as negative control.

To get a broader understanding of EIF4E1 interactions, the protein binding partners of EIF4E1-PTP were analysed by quantitative mass spectrometry. GFP-PTP (Fig 1B) served as a negative control. The pull-downs were done in the presence or absence of 4EIP both forms (S1 Table). Results from procyclic forms were particularly clear, perhaps because they express only the tagged version. We then looked for significantly enriched proteins (for criteria see S1 Table legend). Comparison of EIF4EI-PTP with the GFP-PTP control (Fig 2A) revealed, as expected, that EIF4E1 was associated with 4EIP. *T*. *brucei* 4EIP2 (Tb927.10.11000) was also present in all three replicates and absent in all of the GFP controls; and it also co-purified in the absence of 4EIP, suggesting that it interacts with EIF4E1. Strikingly, the terminal uridylyl transferase TUT3 (Tb927.10.7310) was strongly associated with the EIF4E1 in the presence of 4EIP, but not when 4EIP was absent (Fig 2B). Of the two poly(A) binding proteins, PABP1 was clearly associated with EIF4E1—this might occur via bound RNA but the possibility was not investigated. The association of PABP1 was lower in the absence of 4EIP, but interpretation of this result is somewhat difficult because 4E1-PTP was—as noted above—more abundant when 4EIP was also expressed. PABP2 was significantly enriched with EIF4E1-PTP (+4EIP), but it is also quite abundant in the GFP-PTP controls. Interestingly, two components of the CAF1-NOT deadenylation complex [51], CAF1 and CAF40, were present in all EIF4E1 pull-downs with 4EIP, but not detected with GFP or in the absence of 4EIP. The major scaffold protein NOT1 was also far more abundant with EIF4E1+4EIP than in the other two conditions, but showed below-threshold significance because the yields were variable. Finally, the E3 ligase component SKP1 copurified with EIF4E1 whether or not 4EIP was present. Notably, The only translation factor that was significantly enriched was the ERF1 eukaryotic peptide chain release factor subunit; this was lost when 4EIP was absent. EIF3A was present in one of the three replicates for all three conditions, including GFP. The only protein that was significantly present in the absence of 4EIP, but not in its absence, was the RNA-binding protein HNRNPF/H, which is implicated in control of RNA processing and stability [52]

Results from bloodstream forms were irreproducible since one sample set had much more background than the other two, meaning that most results were not statistically significant (S2A and S2B Fig) Nevertheless, 4EIP2 was reproducibly present and the 4EIP-dependent association of EIF4E1 with TUT3 was confirmed. Once again, members of the CAF1/NOT complex were enriched in a 4EIP-dependent fashion: but this time, NOT5, NOT10 and NOT11 were found.

To re-test the interactions detected by mass spectrometry, co-immunoprecipitations were performed using bloodstream forms. TUT3 was reliably pulled down with 4EIP, and vice versa, whether RNase was added or not (Fig 2C and 2D). Under the conditions used, the proportions of each protein that were co-precipitated were low, but clearly above the background, suggesting that interactions may be transient. As predicted by the mass spectrometry, when we pulled down EIF4E1-PTP using cells expressing V5-tagged EIF3A, no association was detected above the negative control, whether or not 4EIP was present (S2C and S2D Fig). The putative interaction with the NOT complex, judged by co-immunoprecipitation of the exonuclease CAF1, was also weak (S2E Fig) and not reproducible (S2F Fig). Notably, we had previously obtained similar results concerning interactions of 4EIP in bloodstream forms: NOT1 was enriched with 4EIP by mass spectrometry, but no interaction was detected by co-immunoprecipitation [35]. Attempts to detect an interaction of EIF4E1 with tagged NOT1 were thwarted because detection of the (very large) tagged protein was highly unreliable. Although these results are inconclusive, the persistent identification of NOT complex components with EIF4E1-4EIP suggests that 4EIP undergoes transient, low-stoichiometry interactions with the NOT complex.

### TUT3 interacts with 4EIP, but not EIF4E1, in the yeast 2-hybrid assay

To evaluate whether the interaction between TUT3 and 4EIP was direct, yeast 2-hybrid assays were performed, further including the *T*. *brucei* homolog of Dis3L2. Yeast cells could grow on plates lacking adenine and histidine (A-/H-) when both 4EIP and TUT3 were expressed, indicating a mutual interaction; this was more pronounced with the combination of TUT3-AD (activation domain) and 4EIP-BD (DNA-binding domain) (Fig 2E) than *vice versa*. The cells could also survive the selection process to some extent when 4EIP-AD and 4EIP-BD were expressed, suggesting self-interaction. The cells failed to grow with any of the other combinations tested, except for the positive control. The combined results therefore suggest that TUT3 is recruited to the EIF4E1-4EIP complex via 4EIP.

### TUT3 is a cytoplasmic protein that is essential in differentiation-competent bloodstream forms

**Fig 3:**
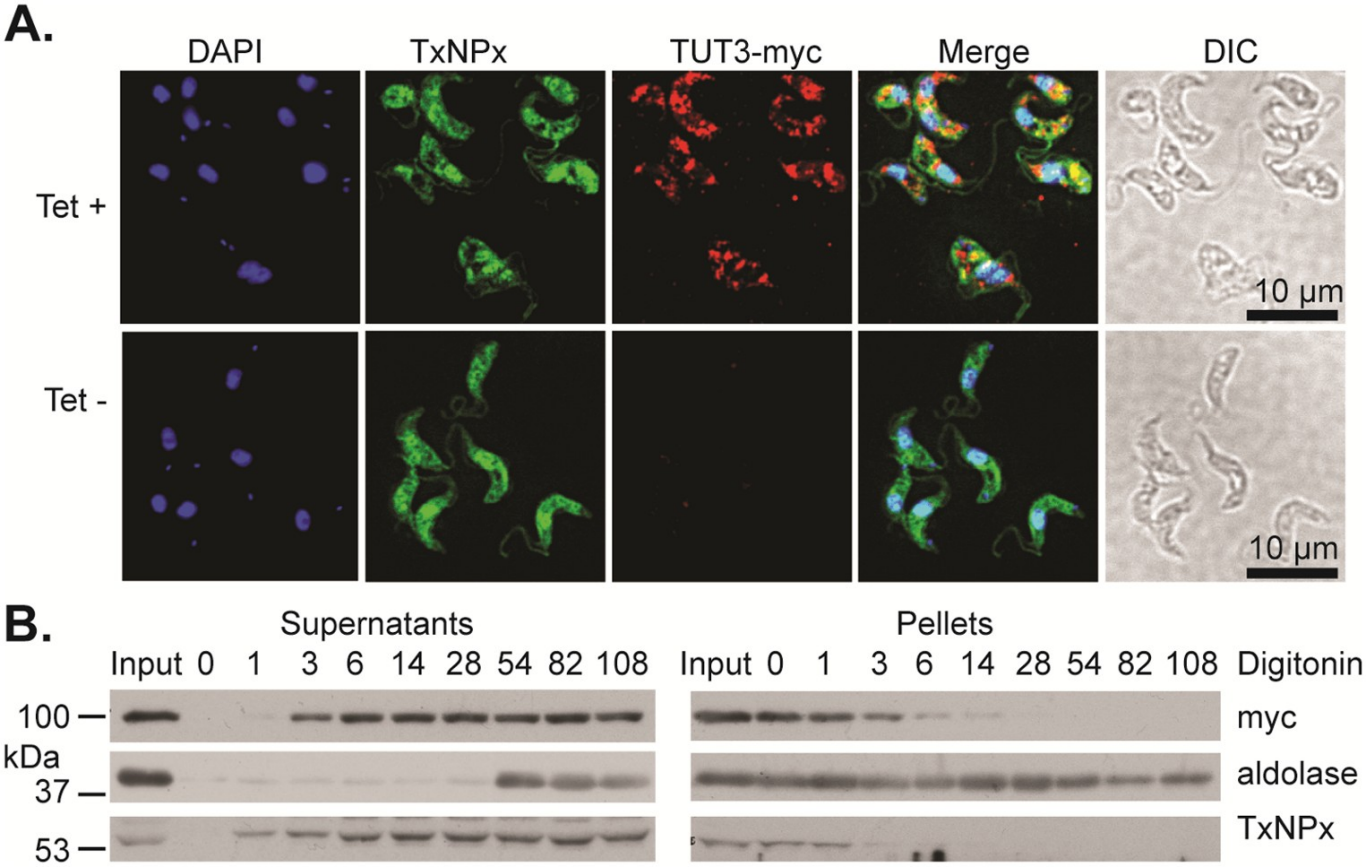
C-terminally tagged TUT3 is not restricted to the mitochondrion. **(A)** Bloodstream-form cells expressing tetracycline-inducible TUT3-myc were stained for tryparedoxin peroxidase (TxNPx), which is cytosolic, for the myc tag, and for DNA (DAPI). DIC—differential interference contrast. In the upper panel, expression of TUT3-myc was induced for 24 h. **(B)** Digitonin fractionation. Cells were permeablised on ice with various concentrations of digitonin, which preferentially permeabilises the plasma membrane. The numbers on the lanes represent the mass of digitonin used per mass of protein (μg/mg) assuming that 5.4 × 10^6^ cells equal 100 mg of protein. "Input" is the total cell lysate. TUT3-myc was released at the same digitonin concentration as TxNPx.

TUT3 could cooperate with 4EIP only if it is in the cytosol, which is indeed the result from high-throughput tagging: both N-terminally and C-terminally GFP-tagged TUT3 were cytosolic [53]. Other trypanosome TUTases are, however, in the mitochondrion. To re-check the location of TUT3, we expressed a C-terminally myc-tagged version in bloodstream forms. (Targeting to the mitochondrial matrix generally requires an N-terminal signal.) By immunofluorescence, TUT3-myc partially co-localised with a cytosolic marker (Fig 3A). Upon digitonin fractionation, TUT3 was also clearly in the cytosolic fraction (Fig 3B).

To examine the function of TUT3, we created procyclic forms in which one copy of *TUT3* bore a sequence encoding an N-terminal V5 tag. RNAi targeting *TUT3* reduced V5-TUT3 expression, but had no effect on growth (S4A Fig). Since there is always residual expression after RNAi, we also attempted to knock out both *TUT3* genes. Multiple attempts to do this, or achieve more effective RNAi, in differentiation-competent bloodstream forms failed. Interestingly, though, we did succeed in Lister 427 bloodstream forms, with no effect on growth (S4B–S4D Fig). This result hints that TUT3 might be involved in aspects of gene expression control that are required only for the survival of fully differentiation-competent trypanosomes.

### Procyclic trypanosomes without 4EIP show reduced expression of *GPEET* mRNA

**Fig 4:**
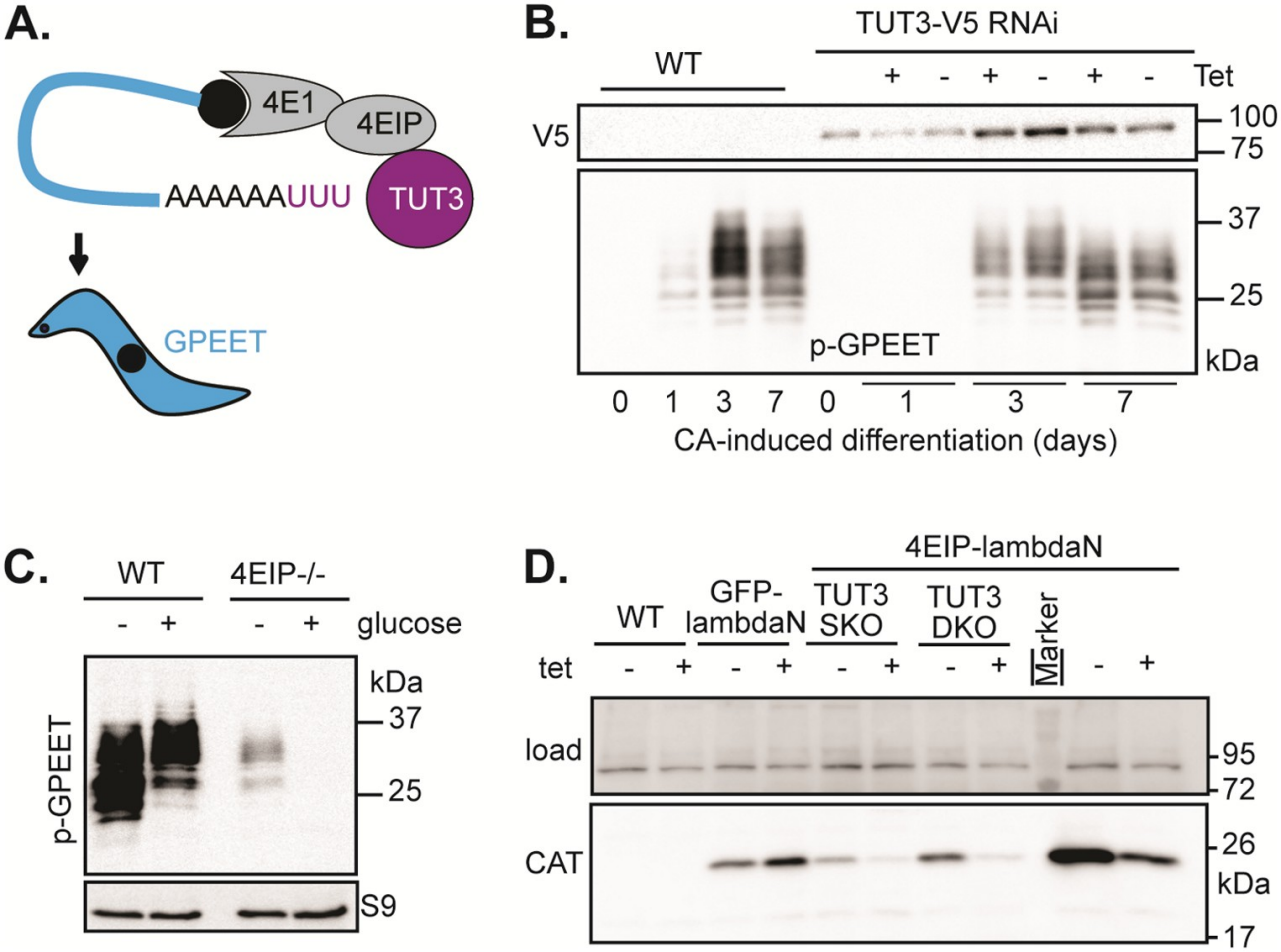
Tethered 4E-Interacting Protein (4EIP) suppresses expression independently of TUT3. **(A)** Schematic representation of *GPEET* procyclin mRNA potentially serving as a substrate for TUT3-mediated uridylation upon recruitment by the EIF4E1/4EIP complex. **(B)** Analysis of phospho (p)-GPEET expression kinetics at the times indicated after *cis*-aconitate (CA)-induced differentiation in WT and TUT3-depleted cells. **(C)** Analysis of p-GPEET expression after adaptation of WT and 4EIP-deficient procyclic forms (PCFs) to 10 mM glucose. **(D)** Expression analysis of chloramphenicol acetyltransferase (CAT) by western blotting after tethering of lambdaN-4EIP- to the 3’UTR of the *CAT* mRNA through a box-B sequence in WT, *TUT3* single-knockout, and *TUT3* double-knockout backgrounds. As a control, lambdaN-GFP was tethered to the CAT mRNA in a WT background.

The results so far suggested that 4EIP might act by recruiting TUT3, resulting in 3’ uridylation and subsequent attack by DIS3L2 and then the exosome. The only uridylated mRNA described for *T*. *brucei* is that encoding the procyclin GPEET [54]. This protein is expressed in the early stages of differentiation of bloodstream forms to procyclic forms, but is later replaced by the EP procyclins [55]. GPEET expression is suppressed by cultivation in the presence of glucose [56] and promoted by addition of glycerol, and regulatory sequences are in the 3’-untranslated region [57, 58]. Non-templated Us in *GPEET* mRNA were detected in both early and late procyclics, but the proportion of mRNAs containing it was not stated, and the role of uridylation in GPEET mRNA decay is not known [54]. To find out whether TUT3 is involved in the regulation of GPEET expression, we induced differentiation of bloodstream forms with TUT3 RNAi by adding *cis*-aconitate and a shift in temperature from 37°C to 27°C. Expression of GPEET was induced in both WT and TUT3-V5 RNAi cells, with a certain delay in the case of the latter (Fig 4A and 4B), while expression of EP procyclin was induced with normal kinetics (S5 Fig). Since, however, RNAi has low efficacy during growth arrest, the results are difficult to interpret. We therefore took another approach. To analyse whether disruption of EIF4E1/4EIP/TUT3 complexes could affect GPEET expression, we compared expression of GPEET on the surface of WT and 4EIP KO procyclic cell lines either in our standard low-glucose medium or after growth in high (10 mM) glucose for three weeks (Fig 4C). Intriguingly, cells lacking 4EIP had strongly reduced GPEET levels compared to WT cells even without growth in high-glucose medium. Nonetheless, as for WT cells, the level of GPEET expression was further reduced in response to the glucose treatment (Fig 4C). The results clearly showed that 4EIP is not required for glucose-induced loss of GPEET expression in procyclic forms.

We therefore decided to look for uridylated *GPEET* mRNA in our procyclic cells. We examined *GPEET* mRNA 3’-ends in normal and glucose-adapted WT procyclic forms by cap removal, circularisation, selective amplification, and DNA sequencing. Poly(A)-tailed *GPEET* mRNAs were identified in 8 of 20 cloned products from low-glucose-grown cells, and only 1 of 20 from the high glucose cells; remaining clones were not *GPEET*. No uridylation was observed (S6 Fig), but deeper sequencing might have revealed it. As an alternative, we searched published RNASeq datasets from strain Lister 427 procyclic forms, also including cells that had been incubated for 30 min with Actinomycin D, since this had been reported to enhance detection of uridylation [54]. These cells clearly expressed *GPEET* as well as *EP* procyclin mRNA, as judged by coding region reads, but examination of 60 reads from the 3’ 100 nucleotides revealed just one possible instance of uridylation. Since the reads were relatively short, we also scanned a publicly available dataset from another project with EATRO1125 procyclic forms (Array Express E-MTAB-10251). The last 100 nt of *EP1* and *GPEET* mRNAs are indistinguishable, but results of coding region searches suggested that *GPEET* mRNAs were more abundant. Of over 2400 sequences from the 3’ 100nt, none had non-coded (U)_4_ tracts. We concluded that uridylation does not play a role in control of *GPEET* in our cell cultures, which have glucose only from the serum.

**Fig 5:**
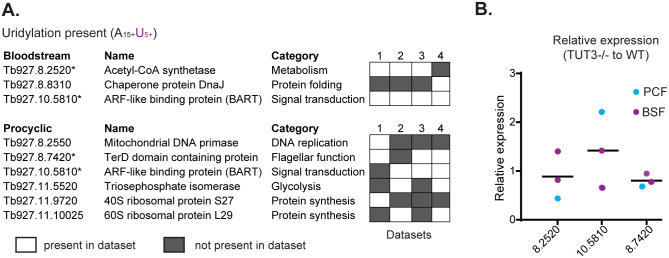
Searching for potential uridylation targets in *Trypanosoma brucei*. (A) *In silico* identification of potential 3’ uridylation targets by screening of RNA sequencing data from wild-type (WT) bloodstream forms (BSFs) and procyclic forms (PCFs) (four data sets each) for sequences containing at least 15 As followed by more than 5 Us. (B) Expression analysis of potential uridylation targets identified by the *in silico* analysis in (A) using quantitative reverse transcription PCR (qRT-PCR).

We also looked for other potential targets of uridylation using four existing RNA-seq datasets from untreated Lister 427 bloodstream forms and procyclic forms (Fig 5A). We were able to identify several mRNAs containing at least 15 As followed by more than 5 Us at their 3’ ends. The levels of three mRNAs that were identified in at least three out of four data sets were compared in WT versus *TUT3* KO cells by RT-qPCR. Levels were similar between WT and *TUT3* KO cells for all three mRNAs analysed (Fig 5B), ruling out a role for TUT3 in degradation of these mRNAs in Lister 427 bloodstream forms.

### TUT3 is not required for 4EIP-mediated repression when tethered to a reporter mRNA

We finally asked whether TUT3 was required in order for 4EIP to repress expression upon tethering to a reporter mRNA. For this, 4EIP fused to a λN peptide was artificially tethered to the 3’ UTR of the mRNA encoding chloramphenicol acetyltransferase (CAT) through a box-B sequence. This was done in WT, *TUT3* single-KO, and *TUT3* double-KO backgrounds. As shown in Fig 4D, 4EIP could exert its repressive functions in absence of TUT3 in long slender bloodstream forms. Rather unexpectedly, tethering of TUT3 to the reporter had no effect on reporter expression (S5B Fig). This was surprising because we might at least have expected the tethered TUT3 to recruit 4EIP to mRNAs. We therefore found no evidence that TUT3 can promote mRNA degradation in bloodstream-form trypanosomes.

### Association of EIF4E1 with mRNAs

**Fig 6:**
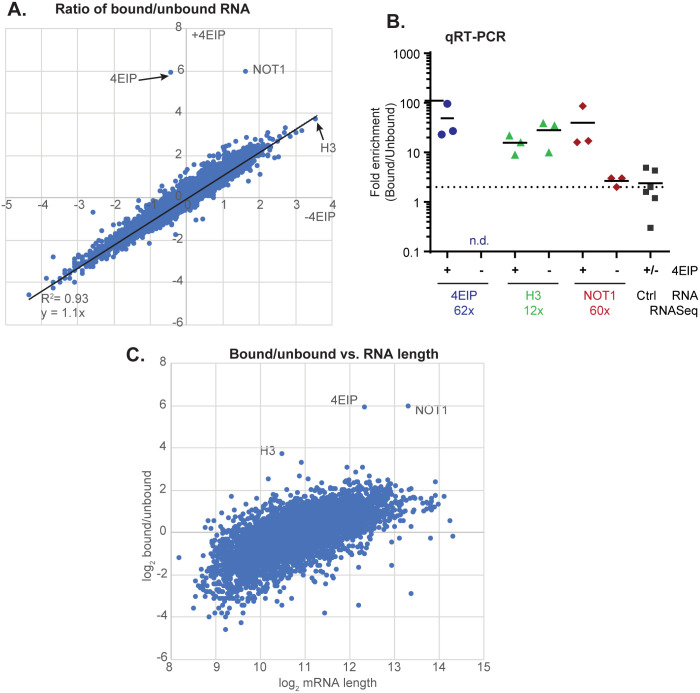
Identification of mRNAs bound to EIF4E1 ± 4E-Interacting Protein (4EIP) in *Trypanosoma brucei*. (A) The mRNAs pulled down with EIF4E1 (± 4EIP) from procyclic forms. The three most strongly enriched mRNAs are indicated. **(B)** Analysis of mRNAs found enriched with EIF4E1-PTP upon RNA sequencing using quantitative reverse transcription PCR (qRT-PCR) with specific primers. **(C)** Ratios of mRNA from EIF4E1-bound and -unbound fractions compared to mRNA length. **(D)** The mRNAs bound to EIF4E1 in procyclic forms were compared with those bound to EIF4E3 in bloodstream forms. The comparison was restricted to mRNAs that show no developmental regulation of abundance or ribosome occupancy.

There is *in vitro* evidence that 4EIP prevents EIF4E1 binding to the cap. If this were true *in vivo*, it might be evident from the EIF4E1-associated transcriptome since in the absence of 4EIP, binding of EIF4E1 to mRNAs should be increased. To investigate this, we performed pull-downs of EIF4E1-PTP in WT and *4EIP* KO backgrounds using procyclic forms, where EIF4E1 is essential. The bound protein was released with TEV protease (which cleaves within the tag), and RNAs in both the bound and unbound fractions were sequenced. Similar amounts of RNA were obtained in both purifications, but since only a single purification step was employed, with no replicates, no major conclusions can be drawn. Moreover, we know that there is less EIF4E1 in *4EIP* KO cells, which makes interpretation of all of the results problematic. We compared the abundances of specific mRNAs in the bound and unbound fractions, in order to find out which mRNAs were specifically bound to EIF4E1 and to see if there were likely to be any differences that would be worth follow-up with more replicates. The results in the presence and absence of 4EIP were almost identical (Fig 6A). There were two notable exceptions: the mRNAs encoding NOT1 and 4EIP, both of which were enriched about 60-fold in the presence of 4EIP. (*4EIP* mRNA is not present in the absence of the gene; the few reads detected must be shared with other genes.) Although this was only a single experiment, the results were overall so similar that adding two more replicates could not possibly increase the number of significantly differently associated mRNAs. We therefore instead confirmed the association of *4EIP*, histone H3 (*HISH3*) and *NOT1* mRNAs in three additional independent pull-downs (Fig 6B). The specific association of EIF4E1 with *4EIP*, *NOT1* and *HISH3* RNAs was superimposed on a background general preference for long mRNAs (Fig 6C). Results from other pull-downs suggest that this might result from relatively non-specific RNA-protein interactions [59].

## Discussion

The aim of this work was to clarify the functions of EIF4E1 both within and outside the complex with 4EIP. As a first step, tagged EIF4E1 was purified from differentiation-competent bloodstream form and procyclic forms and bound proteins were identified. Our results provided no evidence that 4EIP inhibits an *in vivo* translation initiation activity of EIF4E1: no specific associations of EIF4E1 with general translation factors were detected, even in the absence of 4EIP. We did, however, have some hints regarding 4EIP function. We found clear evidence for association of 4EIP with the terminal uridylyl transferase TUT3, although its role is uncertain, and cells lacking 4EIP also had decreased levels of EIF4E1 protein. The protein 4EIP2 was associated with trypanosome EIF4E1 independently of 4EIP, suggesting binding to EIF4E1 as already indicated for *Leishmania* [28]. Both N-and C-terminally tagged versions of *T*. *brucei* 4EIP2 are in the cytoplasm [53]; high-throughput RNAi detected no defect for 4EIP2 depletion [49], but the degree of depletion was of course not assessed. Pull-down results in *Leishmania* suggested that about a tenth of the EIF4E1 was 4EIP2-associated. Since 4EIP2 was not detected in our previous 4EIP pull-downs [35], and the interaction most likely occurs via the same motif, 4EIP2 and 4EIP probably compete for EIF4E1 binding. It is possible that when 4EIP1 is absent, it is replaced by 4EIP2; this will depend on whether other EIF4Es are also competing for 4EIP2, and on the relative abundances of the different proteins.

Our conclusions differ from those that have been drawn for *Leishmania* EIF4E1. An interaction of *Leishmania* EIF4E1 with EIF3A was seen by mixing two cell lysates, one from cells over-expressing streptavidin-binding-peptide (SBP)-tagged- EIF3A, and the other from cells overexpressing untagged EIF4E1. EIF4E1 was pulled down with SBP- EIF3A, but not with SBP-luciferase [33]. Second, the two proteins interacted in a well-controlled yeast 2-hybrid assay [33]. Also, addition of an N-terminal fragment of *Leishmania* 4EIP to cell lysates inhibited binding of EIF4E1 to m^7^-GDP [34]; and it is known that EIF4E1 increases in abundance in the intracellular amastigote stage. Together, these results led the authors to conclude that EIF4E1 could be a translation initiation factor in *Leishmania* amastigotes. We had therefore expected that elimination of 4EIP from trypanosomes—which are much easier to manipulate genetically than *Leishmania*—might reveal a cryptic initiation activity, as judged by association with other initiation factors or with mRNA. However, our mass spectrometry results yielded no evidence for association of EIF4E1 with general translation factors. Moreover, independent precipitation of EIF4E1-PTP, followed by western blotting, revealed no co-purification of EIF3A even if 4EIP was absent. In our previous tethering assays, EIF4E3, 4, 5 and 6 all were able to stimulate expression of the bound reporter RNA [36], but in bloodstream forms at least, tethered EIF4E1 has no effect, even on a 4EIP null background [35]. Of course it is possible that in the absence of 4EIP, 4EIP2 takes over and again prevents EIF4E1 cap binding; or that EIF4E1 may act as an initiation factor in other parts of the life cycle. But nevertheless, together these results provide no indication that EIF4E1 is able to drive translation initiation in *T*. *brucei* bloodstream forms or procyclic forms. Instead, we suggest that EIF4E1 supports repression by 4EIP.

Results from ribosome profiling have shown that in yeast, some protein complexes assemble co-translationally [60]. EIF4E1 and 4EIP form a stable complex and we found that purification of EIF4E1-PTP resulted in extremely strong enrichment of *4EIP* mRNA. 4EIP interacts with EIF4E1 via the 4EIP N-terminus. We therefore suggest that *4EIP* mRNA is be connected to EIF4E1-PTP via the nascent 4EIP polypeptide. Since the EIF4E1-PTP was purified via a C-terminal tag, the result might mean that there is a small pool of EIF4E1-PTP that is free for nascent 4EIP binding, and not bound by pre-existing 4EIP or 4EIP2. However, levels of EIF4E1 were drastically reduced in cells lacking 4EIP, suggesting that the mutual interaction is required for EIF4E1 protein stability—a function that 4EIP2 evidently cannot replace. The IBAQ values, although not reliable for absolute protein quantification, suggest roughly equimolar co-purification of 4EIP with EIF4E-PTP, and ten times less 4EIP2 (S1 Table). These combined results therefore suggest that stabilisation of EIF4E1 by 4EIP may be initiated by co-translational folding of the two proteins.

Drosophila GIGYF2—like 4EIP—is essential for repression by 4EHP, acting in part through recruitment of the NOT deadenylation complex [61]. Purification of 4EIP or—in this paper, EIF4E1 with associated 4EIP, has reproducibly yielded results suggesting the association of small amounts of NOT complex, hinting that 4EIP might repress via deadenylation in trypanosomes, as well. However, the complex subunits that were reproducibly present were different each time, and by co-immunoprecipitation, the amounts of tagged CAF1 were so small that they were difficult to distinguish from background. Thus if this interaction occurs, it must be transient or weak, involving only a small proportion of each complex. In procyclic forms, the *NOT1* mRNA was very strongly associated with EIF4E1, in a 4EIP-dependent fashion. We do not know what this result means: whether the mRNA is purified via the nascent polypeptide, or via direct RNA binding. In bloodstream forms, the *NOT1* mRNA was not enriched with 4EIP [35]. Thus the relationship between EIF4E1, 4EIP and the NOT complex remains enigmatic.

When we saw the interaction between 4EIP and TUT3, we immediately thought that 4EIP-mediated suppression might act through TUT3-mediated 3’-uridylation, followed by 3’ digestion by DIS3L2. Targets of uridylation are difficult to identify based on their (presumed) unstable nature. Our investigations concerning this were inconclusive. We were unable to confirm *GPEET* mRNA as a target of 3’-terminal uridylation, and the abundances of some other potential targets were unaffected in cells lacking TUT3. TUT3 also was not essential for the suppressive activities of 4EIP, at least when tethered to a reporter mRNA. The tethering experiments were, however, all done in differentiation-incompetent bloodstream forms, in which EIF4E1, 4EIP and TUT3 are not essential. We could not obtain differentiation-competent bloodstream forms, or procyclic forms, that completely lack TUT3, or lines with effective RNAi, so could not investigate this further. These results too are inconclusive, but the possibility remains that TUT3 might destabilise some mRNAs that promote differentiation in growing cells, and might also cooperate with the 4EIP-EIF4E1 complex.

## Materials and methods

### Trypanosome culture and differentiation

All experiments were done in pleomorphic EATRO1125 strains ectopically expressing the *Tet* repressor. Bloodstream forms were cultured at 37°C in HMI-9 medium (supplemented with 10% (v/v) fetal calf serum (FCS), 1% (v/v) penicillin/streptomycin solution (Labochem international, Germany), 15 μM L-cysteine, and 0.2 mM β-mercaptoethanol) in the presence of 5% CO_2_ and 95% humidity and their density was maintained below 1.0 × 10^6^ cells/mL unless stated otherwise. Procyclic forms were cultured at 27°C in MEM-Pros media (supplemented with 10% (v/v) FCS, 3.75 mg hemin, and 1% (v/v) penicillin/streptomycin solution (Labochem international, Germany)), and their density was maintained between 5 × 10^5^ and 5 × 10^6^ cells/mL. Cell densities were determined using a Neubauer chamber.

To induce bloodstream form to procyclic form differentiation, cells grown at 1 × 10^6^ cells/mL in HMI-9 medium were incubated with 6 mM *cis*-aconitate at 27°C for 24 h. On the following day, the medium was exchanged for MEM and the cells were cultured at 27°C afterwards.

### Immunofluorescence microscopy

Bloodstream forms at a density of 1.0 × 10^6^/mL were collected at 950 × *g* and washed with PBS, then fixed using 4% para-formaldehyde for 20 min at RT. Fixed cells were left overnight at 4°C to settle down on a chambered poly-L-lysine treated microscope slide. The cells were permeabilised by 0.2% Triton X-100 for 20 min and subsequently washed three times using PBS in 5 min intervals. Blocking was done for 20 min using 0.5% gelatin. The cells were incubated with primary antibodies (myc, 1:300; TxNPx, 1:1000; aldolase, 1:300) for 1 h and subjected to three more washing steps. The cells were incubated with secondary antibodies (1:500) coupled to fluorophores for 1 h, and washed twice before treatment with DAPI at 500 ng/mL for 15 min. The cells were washed twice and mounted using 90% glycerol in PBS. Images were acquired using an Olympus IX81microscope and analysed with Olympus Xcellence or Image J software. This experiment was done once.

### Genetic manipulation of trypanosomes

Gene knockout was achieved by replacing the open reading frames (ORFs) of the target gene with ORFs encoding blasticidin and puromycin resistance genes, which integrate by homologous recombination. Double knock-out cells were selected by growing transfectants in both 5 μg/mL blasticidin and 0.2 μg/mL puromycin for at least two weeks before downstream analyses. Similarly, tagging with V5, myc or PTP tags was achieved by integration through homologous recombination in 5’- or 3’-UTRs for N- or C-terminal tagging of the protein of interest, followed by selection of transfectants with the respective drug. Selection of EATRO1125 clones was done using half the drug concentrations. The primers and plasmids used in this study can be found in S3 Table.

### DNA extraction

DNA was extracted from approximately 3 × 10^8^ cells as follows. The cell pellet was resuspended and the cells were lysed using 0.5 mL EB buffer (10 mM Tris-HCl pH 8.0, 10 mM NaCl, 10 mM EDTA). RNA was digested using 25 μg/mL of RNaseA (Sigma-Aldrich) at 37°C for 30 min. Proteins were precipitated using 1.5 M ammonium acetate and centrifuging at 9,391 × *g* for 5 min. Isopropanol was added to the supernatant in a 1:1 ratio and centrifuged at 15,871 × *g* for 15 min at 4°C. The pellet was washed with 75% ethanol followed by centrifugation at 15,871 × *g* for 5 min and dried before resuspending in 50 μL TE buffer (10 mM Tris pH 7.5, 1 mM EDTA pH 8.0). PCR was done using Taq Polymerase according to the manufacturer’s instructions (New England Biolabs).

### Immunoprecipitations

All co-immunoprecipitations were done at least twice. For cell harvest, 2 × 10^9^ (for MS/RNA-IP) or 1 × 10^8^ (for WB) trypanosomes at a concentration of 5 × 10^5^ cells/mL (BSF) or 5 × 10^6^ cells/mL (PCF) were centrifuged at 3,000 rpm for 13 min at 4°C. The pellet was resuspended in 50 mL of ice-cold PBS and centrifuged at 2,300 rpm for 8 min. After removal of the supernatant, the pellet was snap-frozen in liquid nitrogen and stored at -80°C until further processing. All of the following steps were at 4°C, unless stated otherwise. Cell pellets were thawed on ice and resuspended in 0.5 mL of lysis buffer (20 mM Tris [pH 7.5], 5 mM MgCl_2_, 1 mM DTT, 0.05% IGEPAL, 100 U/mL RNasin, 10 μg/mL aprotinin, and 10 μg/mL leupeptin). For releasing protein contents, the cells were passaged 20× through a 21G×1½” needle and 20× through a 27G×¾ needle using a 1 mL syringe. In order to pellet the cell debris, samples were centrifuged at 10,000 × *g* for 15 min, and the supernatant was transferred to a fresh tube. The salt concentration was then adjusted to 150 mM KCl. Magnetic beads (Dynabeads^™^ M-280 Tosylactivated, Thermo Fisher Scientific) coupled to rabbit IgG were adjusted by three sequential washes with wash buffer (20 mM Tris [pH 7.5], 5 mM MgCl_2_, 1 mM DTT, 0.05% IGEPAL, 100 U/mL RNasin, 150 mM KCl). Depending on the cell number, 10–100 μL of the beads were then added to each sample. To allow binding, cell lysate and beads were incubated for 1–2 h at 4°C while rotating (20 rpm).

The beads were boiled directly in Laemmli buffer for 10 min at 95°C and analysed by western blotting (see below).

### Harvest for mass spectrometry of EIF4E1-associated proteins

Pull-downs were performed with 10^9^ bloodstream forms or procyclic forms according to the procedure described above under "Immunoprecipitations". The beads were washed four times with wash buffer, after which bound proteins were released by TEV cleavage. For this, 20 μL of wash buffer and 1 μL of recombinant TEV protease (1 mg/mL) were incubated with the beads for 90 min at 20°C. For removal of His-tagged TEV, IgG magnetic beads were concentrated on one side, the supernatant was transferred to a fresh tube, and 10 μL of equalization buffer (200 mM sodium phosphate, 600 mM sodium chloride, 0.1% Tween-20, 60 mM imidazole, pH 8.5), as well as 30 μL of Ni-NTA-magnetic beads were added and incubated with the samples for 30 min at 20°C while rotating. Ni-NTA magnetic beads were retained by a magnetic stand and the supernatant was collected and stored in Laemmli buffer at -80°C.

Eluted proteins were separated on a 1.5 mm NuPAGE^™^ Novex^™^ 4–12% Bis-Tris protein gel (Thermo Fisher Scientific) until the running front had migrated roughly 2 cm, after which the gel was stained with Coomassie blue and destained with destaining solution (10% acetic acid, 50% methanol in H_2_O). Three areas per lane were cut and analysed by Nanoflow LC-MS2 analysis with an Ultimate 3000 liquid chromatography system directly coupled to an Orbitrap Elite mass spectrometer (both Thermo-Fischer, Bremen, Germany). MS spectra (m/z 400–1600) were acquired in the Orbitrap at 60,000 (m/z 400) resolution. Fragmentation in CID cell was performed for up to 10 precursors. MS2 spectra were acquired at rapid scan rate. Raw files were processed using MaxQuant (version 1.5.3.30; J. Cox, M. Mann, Nat Biotechnol 2008, 26, 1367) for peptide identification and quantification. MS2 spectra were searched against the TriTrypDB-8.1TREU927-AnnotatedProteins-1 database (containing 11567 sequences). Data were analysed quantitatively and plotted using Perseus software (Version 1.6.6.0) [62]. Raw data are available in the PRIDE database with the accession number PXD025913.

For the procyclic trypanosomes, three preparations were analysed at the same time as the GFP-PTP control. For bloodstream forms, one purification was done (A) then repeated twice more at the same time as the procyclic experiment.

### Harvest for isolation or EIF4E1-bound RNA

Unbound samples were collected for RNA extractions, three volumes of peqGOLD TriFast^™^ FL reagent were added, and samples were stored at -80°C until further processing. The beads were washed four times with wash buffer and proteins with bound RNAs were released by incubation with 5 μL recombinant TEV protease (1 mg/mL) in 250 μL of wash buffer for 90 min at 20°C. For collecting the elution fractions, the beads were concentrated on one side of the tube, the supernatant was transferred to a fresh tube, three volumes of peqGOLD TriFast^™^ FL reagent were added, and samples were stored at -80°C until further processing.

### RNA isolation

Samples frozen at -80°C in TriFast reagent were thawed and incubated for 5 min at room temperature to ensure complete dissociation of ribonuclear complexes. Afterwards, 200 μL of chloroform were added per sample, and the tubes were shaken vigorously by hand for 15 s. Subsequent to 3 min of incubation at room temperature, samples were centrifuged at 12,000 rpm for 10 min at 4°C. Following centrifugation, the aqueous phase was transferred to a fresh tube, and 7 μL of glycogen solution (10 mg/mL) were added, as well as 500 μL of isopropanol. The samples were mixed briefly by vortexing and incubated at room temperature for 10 min, before being subjected to another centrifugation step at 12,000 rpm for 15 min at 4°C. The pellet was subjected to two sequential washing steps with 70% and absolute ethanol, respectively. Afterwards, the supernatant was removed and the pellet was dried at room temperature for 10 min. Purified RNA was then dissolved in nuclease-free water by incubation at 50°C for 5 min, and the RNA concentration was determined spectrophotometrically.

This experiment was done once each for procyclic forms expressing 4E1-PTP in the presence or absence of 4EIP.

### Analysis of circular RNAs

For this purpose, 1 × 10^8^ procyclic forms were harvested after growth with or without glucose, and total RNA was extracted according to the standard protocol. Afterwards, 30 μg of RNA were treated with 20 U of DNase I in 200 μL in presence of RNaseIN for 30 min at 25°C. The reaction was terminated by phenol-chloroform extraction and ethanol precipitation of the RNA. The pellet was dissolved in 32 μL of RNase-free water and incubated with 100 pmol of an oligonucleotide complementary to the first 15 nt of the spliced leader (5′ TCTAATAATAGCGTT 3′) at 37°C for 5 min. RNase H buffer was added to reach a concentration of 1×, along with 5 U of RNase H, and the reaction was then incubated for 1 h at 37°C. The RNA was then purified and precipitated with ethanol according to the standard protocol. Subsequently, 10 μg of RNA were circularised by incubation at 16°C for 16 h in a reaction volume of 400 μL containing 40 U of T4 RNA and 80 U of RNaseIN. For reverse transcription, ∼2 μg of RNA were incubated with 50 pmol of GPEET-specific reverse primer, 200 U SuperScript III RT (Invitrogen), and 40 U RNase Inhibitor in a 20 μL reaction volume.

PCR amplification was performed in a 50 μL reaction using 1 μL of cDNA (5%), 10 pmol each of forward and reverse primers, 2.5 U Taq DNA polymerase (30 s at 95°C, 30 s at 52°C, and 45 s at 72°C). The PCR products were gel-purified and cloned into EamI-digested p2T7 vector. Clones were subjected to blue-white selection and analysed by sequencing. 20 clones were sequenced under each condition.

### Expression analysis by RT-qPCR

For this, cDNA was synthesized from unbound and bound fractions of RNA-immunoprecipitations using the Maxima First Strand cDNA Synthesis Kit (Thermo Scientific) according to the manufacturer’s instructions. Afterwards, expression of the mRNAs of interest was analysed in triplicates by qPCR using the Luna^®^ Universal qPCR Master Mix and specific primers (S3 Table). Data were analysed by the 2^-ΔΔCt^ method and plotted with GraphPad Prism software (version 6).

### RNA sequencing and data analysis

RNA-seq was done at the CellNetworks Deep Sequencing Core Facility at the University of Heidelberg. For library preparation, NEBNext Ultra RNA Library Prep Kit for Illumina (New England BioLabs Inc.) was used. The libraries were multiplexed (6 samples per lane) and sequenced with a Nextseq 550 system, generating 75 bp single-end sequencing reads. Data are deposited as E-MTAB-10453.

The quality of the data was checked using FastQC. After primer removal using Cutadapt, the data were aligned to the TREU 927 reference genome using custom scripts, as described previously [63, 64]. Briefly, reads were trimmed using CutAdapt [65], and aligned using Bowtie2 [66] allowing up to 2 mismatches. For manual analysis, a new dataset of 84nt reads from EATRO1125 procyclic forms, which had been generated for another project, was used. 2400 sequences spanning last 100nt of the EP1 or GPEET 3’-UTRs (which are indistiguishable in that region) were manually retrieved by searching for matches to "CAAAGGAATG" or "GCTTAAAATTATG" (antisense to the 3’-UTR). The sequences were then manually examined for uncoded strings of 4 As, and none were found. A search for EP1 and GPEET-specific sequences further upstream revealed a ratio of approximately 1:2.

### *In silico* identification of uridylation targets

Identification of potentially uridylated transcripts was performed RNA sequencing data from four sets of bloodstream form and procyclic form data each (E-MTAB-3793; E-GEOD-79208; E-MTAB-8062; E-MTAB-7996; E-MTAB-3335). The sequence reads, which were 75 nt on average, were analysed for the presence of at least 15 As followed by > 5 Us. Afterwards, the 3’ UTRs preceding the 3’ uridylated transcripts served to identify the corresponding transcript through BLAST search in the TriTryp database.

### Digitonin fractionation

10^8^ cells were collected by centrifugation (950 × *g*, 8 min) and washed three times in trypanosome homogenization buffer, THB (25 mM Tris-Cl [pH 7.8], 1 mM EDTA, 150 mM NaCl, 0.3 M sucrose). The washes consisted of centrifugation (950 × *g*, 3 min, 4°C) and resuspension by gentle agitation of the tube instead of pipetting. The cells were eventually resuspended in 100 μL of THB containing 2 μg/mL leupeptin, 1 mM DTT and always placed on ice. Using a stock of 0.9 mg/mL of digitonin (dissolved in THB 95°C, 5 min), aliquots of different digitonin concentrations were prepared and their volumes adjusted to be similar to each other and set at room temperature. 10^7^ cells (10 μL) were pipetted directly into the digitonin aliquots (at most, three tubes at a time) and incubated for 4 min at 25°C. Subsequently, the tubes were centrifuged (15,871 × *g*, 1.5 min, 4°C) and the supernatants and pellets separated and dissolved in laemmli buffer (125 mM Tris-HCl pH 6.8, 4% SDS, 15 mM EDTA, 10% β-mercaptoethanol, 20% glycerol, 0.1% bromophenol blue). The samples were separated on a 10% SDS-PAGE gel and analysed by western blotting (see above).

### Yeast-2-hybrid assays

For testing direct protein-protein interactions, the Matchmaker Yeast Two-Hybrid System (Clontech) was used according to the manufacturer’s instructions. To that end, ORFs of interest (TUT3, 4EIP, and Dis3L2) were PCR-amplified from genomic DNA and cloned into both pGBKT7 and pGADT7 plasmids. Prey and bait plasmids were co-transformed pairwise into AH109 yeast strains, and selected initially on double drop-out (DDO) plates (i.e., SD medium lacking Trp and Leu) or quadruple drop-out (QDO) plates (i.e., lacking Trp, Leu, His and Ade). Growth on QDO plates indicated positive interactions. The interaction between p53 and SV40 large T-antigen and the combination of LaminC and SV40 large T-antigen served as positive and negative controls, respectively. Western blotting was used to confirm expression of c-myc-tagged BD-domain proteins and HA-tagged AD-domain proteins.

### Protein detection by western blotting

For western blotting, 1–5 × 10^6^ cells were lysed in 1× Laemmli buffer, separated by 8–12% SDS-PAGE, and processed as described elsewhere [67]. The following antibodies were used for specific protein detection: anti-4E1 serum (1:2,000, rabbit; kind gift from Osvaldo de Melo Neto), anti-S9 serum (1:20,000, rat; loading control), anti-myc (1:5,000, mouse; Y2H), anti-p-GPEET (1:2,000, mouse, Cedarlane; western blotting), anti-V5 (1:2,000, mouse, Biorad; western blotting), anti-PAP (1:20,000, rabbit, Sigma; western blotting), anti-aldolase serum (1:50,000, rabbit; western blotting), anti-EP (1:2,000, mouse, Cedarlane; western blotting), and anti-CAT serum (1:2,000, rabbit), anti-rabbit IgG (pull-downs).

## Supporting information

S1 Fig EIF4E1 expression levels. (A) Bloodstream and procyclic forms (BSF and PCF, respectively) were collected at the numbers indicated and analysed for expression of EIF4E1 by western blotting with specific antibodies. The antibody used also variably detects some background bands of unknown identity. (B) Full blots corresponding to Fig 1B. The unmanipulated image is at the top, and on with increased contrast below. The bottom panel is detection of 4EIP-myc.
(PDF)

S2 Fig EIF4E1 interactions in bloodstream forms. **(A)** PTP-tagged GFP and EIF4E1 were pulled down from bloodstream forms for comparison of bound proteins using quantitative mass spectrometry. The statistical significances were low becuse one replicate had many more proteins than the other two. **(B)** PTP-tagged EIF4E1 was pulled down from bloodstream forms for comparison of bound proteins in WT and 4EIP knockout backgrounds using quantitative mass spectrometry. **(C)** PTP-tagged EIF4E1 was pulled down from 1 × 10^8^ bloodstream forms (BSFs) of *Trypanosoma brucei*. Enrichment of V5-tagged EIF3A in the different fractions was analysed by western blotting. A very faint band is seen in the elutions, migrating slightly slower than the V5-EIF3A input, but it is present equally in the presence and absence of 4E1-PTP. The loading is indicated underneath the lanes, as well as the relative amounts of V5-tagged EIF3A, obtained by densitometry and adjusted for loading. **(D)** Same experiment as described in **(C)**, including cells lacking 4EIP. The very faint background band at the position of V5-EIF3A is not affected. **(E, F)** Two representative replicates of pull-downs of PTP-tagged EIF4E1 from bloodstream form *T*. *brucei* parasites, followed by detection of V5-CAF1 by western blotting. 1% each of the input and unbound fractions were loaded. Details, including loading, are as in C and D. In (E) about 1% of the V5-CAF1 was co-precipitated; in (F) co-precipitation was minimal.
(PDF)

S3 Fig Yeast 2-hybrid controls. Western blotting to show expression of fusion proteins in the yeast used in Fig 2E.
(PDF)

S4 Fig Effects of TUT3 depletion or elimination. **(A)** Knockdown of *TUT3* was induced in Lister 427 procyclic forms (PCFs) cultured in presence of tetracycline (+), with wild-type (WT) cells and RNAi cells cultured in absence of tetracycline serving as controls. The cell numbers were determined over the course of 5 days (upper panel), and knockdown efficiency was determined by western blotting (lower panel). **(B)** Primer pairs used for amplifying a 260 bp fragment within the *TUT3* ORF (i) or a fragment spanning the 5’-UTR and the ORF (ii). Primers for amplification of the *PUF3* gene were used as a control (iii). **(C)** Diagnostic polymerase chain reaction for the *TUT3* gene. Each PCR contained primers to amplify *PUF3* (purple, green, band iii) as a positive control. Product (i) was obtained from a primer pair (blue, cyan) that hybridised within the *TUT3* open reading frame. Product (ii) was obtained using one primer that targets the ORF (orange) and another (red) that binds upstream of *TUT3* to a sequence absent from the knockout plasmid. **(D)** The growth of monomorphic bloodstream forms with knockout of either a single copy or both copies of the TUT3 coding sequence (sKO and HKO, respectively) was monitored over the course of 5 days.
(PDF)

S5 Fig Differentiation of TUT3-depleted cells and effect of tethering. **(A)** Differentiation-competent *T*. *brucei* (EATRO1125 strain) with one V5-tagged TUT3 gene and RNAi targeting *TUT3* were grown to a maximum density of 2 × 10^6^ cells/mL. 6 mM *cis*-aconitate (CA) was then added, and the temperature was reduced to 27°C to induce differentiation. Cultures were grown with or without tetracycline, with the drug being included for 24 h before addition of *cis*-aconitate. For each time point, 5 × 10^6^ cells were collected for western blot measurement of V5-TUT3, Ep procyclin, and a control rpotein, ribosomal protein S9. **(B)** Tethering of lambdaN-TUT3-myc has no effect on a box-B-containing reporter mRNA encoding chloramphenicaol acetyltransferase (CAT). Expression of lambdaN-TUT3-myc was induced for 24h then CATwas measured by Western blotting. A protein that cross-reacts with the anti-CAT antibody served as a loading control.
(PDF)

S6 Fig Sequencing of poly(A)-tailed GPEET mRNAs. *GPEET* mRNA 3’-ends in normal and glucose-adapted WT procyclic forms (PCFs) were examined by cap removal, circularization, selective amplification, and DNA sequencing. Identified poly(A)-tailed *GPEET* mRNAs from low- and high-glucose-grown cells are shown; 20 sequences were analysed each.
(PDF)

S1 Table Proteins associated with EIF4E1 in absence and presence of 4EIP. A detailed legend is on the first sheet of the Table.
(XLSX)

S2 Table mRNAs associated with EIF4E1 in procyclic forms, in the absence and presence of 4EIP. A detailed legend is on the first sheet of the Table.
(XLSX)

S3 Table Plasmids and oligonucleotides. (XLSX)
