# Supplementary figures and images for: The EIF4E1-4EIP cap-binding complex of Trypanosoma brucei interacts with the terminal uridylyl transferase TUT3
Source: PLoS One. 2021 Nov 22;16(11):e0258903. doi: 10.1371/journal.pone.0258903 (PMC8608314; doi:10.1371/journal.pone.0258903)

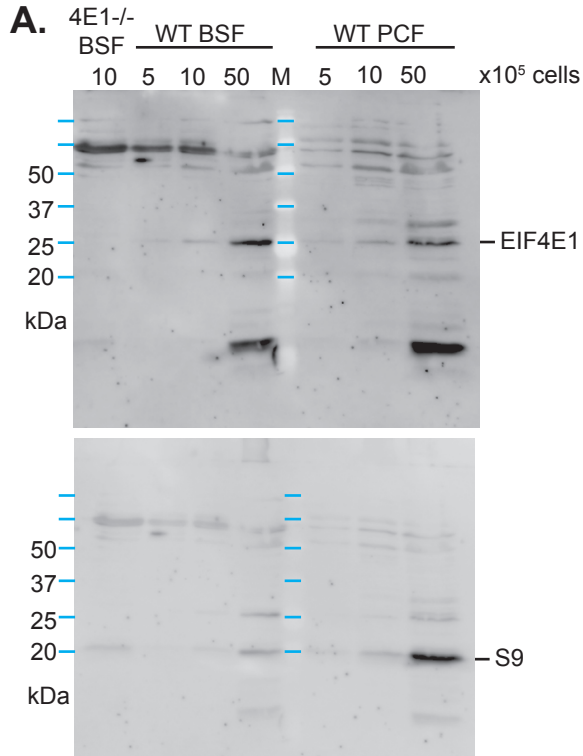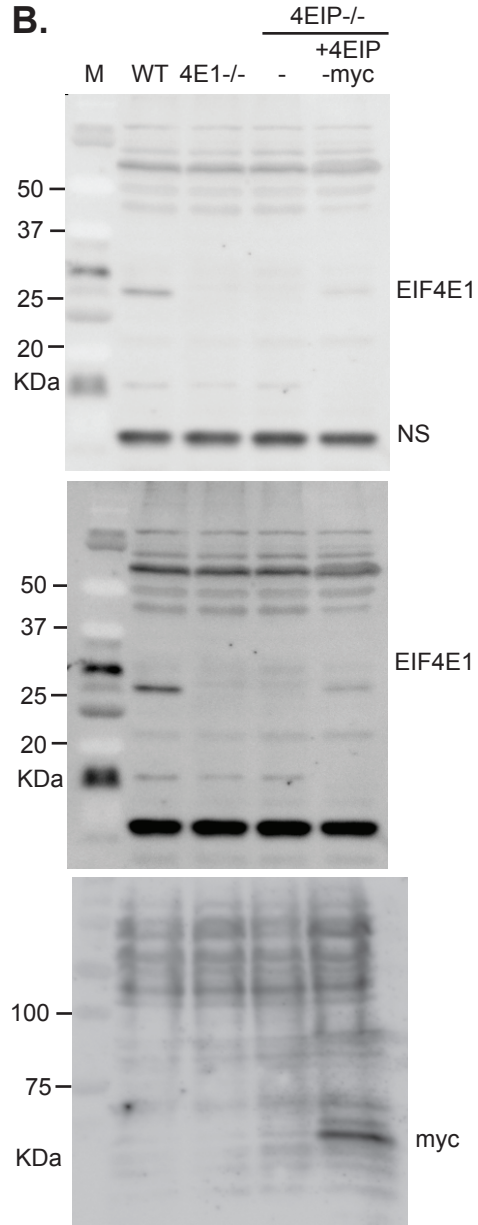

Supplement: S1 Fig — (A) Bloodstream and procyclic forms (BSF and PCF, respectively) were collected at the numbers indicated and analysed for expression of EIF4E1 by western blotting with specific antibodies. The antibody used also variably detects some background bands of unknown identity. (B) Full blots corresponding to Fig 1B. The unmanipulated image is at the top, and on with increased contrast below. The bottom panel is detection of 4EIP-myc. (PDF) [file pone.0258903.s001.pdf]

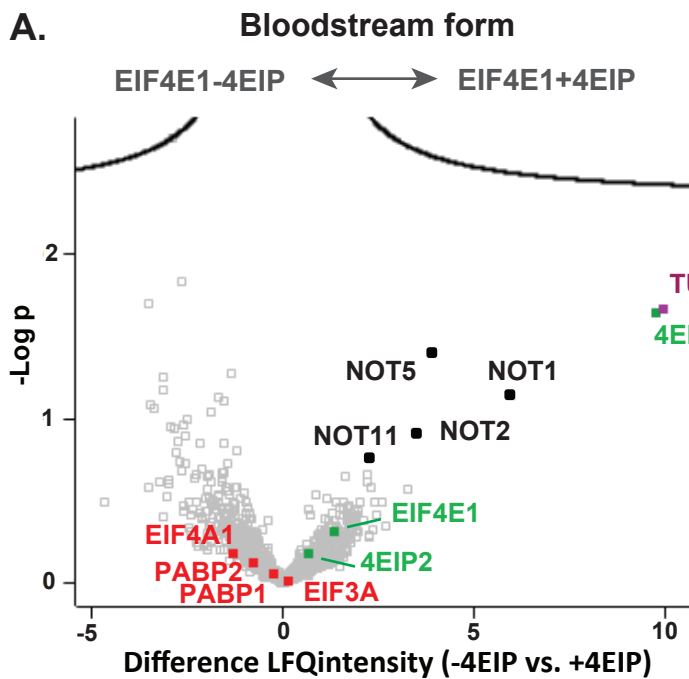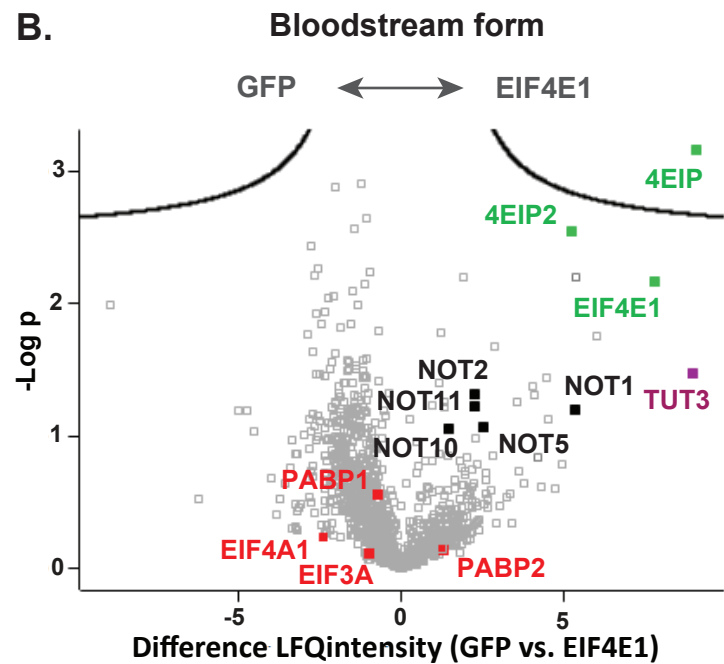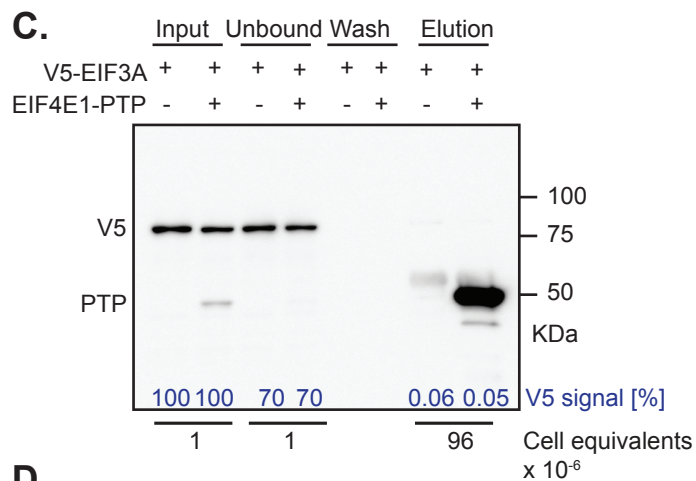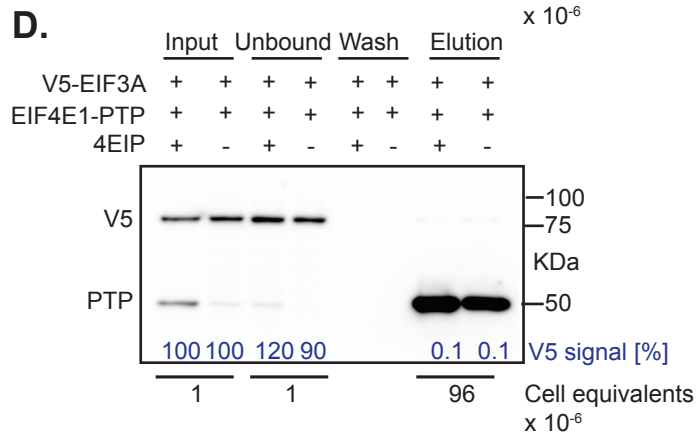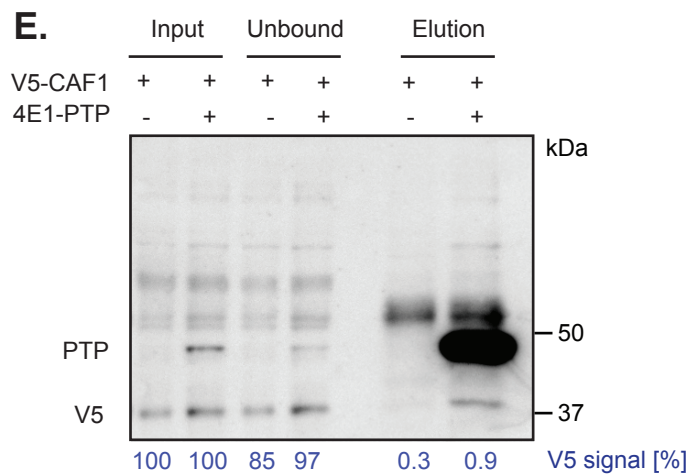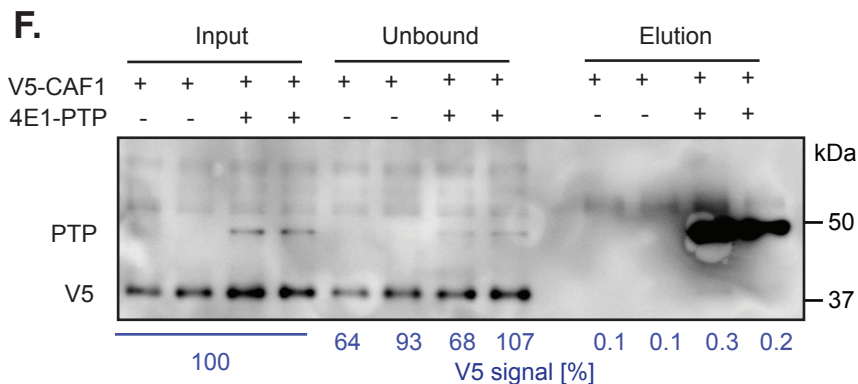

Supplement: S2 Fig — (A) PTP-tagged GFP and EIF4E1 were pulled down from bloodstream forms for comparison of bound proteins using quantitative mass spectrometry. The statistical significances were low becuse one replicate had many more proteins than the other two. (B) PTP-tagged EIF4E1 was pulled down from bloodstream forms for comparison of bound proteins in WT and 4EIP knockout backgrounds using quantitative mass spectrometry. (C) PTP-tagged EIF4E1 was pulled down from 1 × 10^8^ bloodstream forms (BSFs) of Trypanosoma brucei. Enrichment of V5-tagged EIF3A in the different fractions was analysed by western blotting. A very faint band is seen in the elutions, migrating slightly slower than the V5-EIF3A input, but it is present equally in the presence and absence of 4E1-PTP. The loading is indicated underneath the lanes, as well as the relative amounts of V5-tagged EIF3A, obtained by densitometry and adjusted for loading. (D) Same experiment as described in (C), including cells lacking 4EIP. The very faint background band at the position of V5-EIF3A is not affected. (E, F) Two representative replicates of pull-downs of PTP-tagged EIF4E1 from bloodstream form T. brucei parasites, followed by detection of V5-CAF1 by western blotting. 1% each of the input and unbound fractions were loaded. Details, including loading, are as in C and D. In (E) about 1% of the V5-CAF1 was co-precipitated; in (F) co-precipitation was minimal. (PDF) [file pone.0258903.s002.pdf]

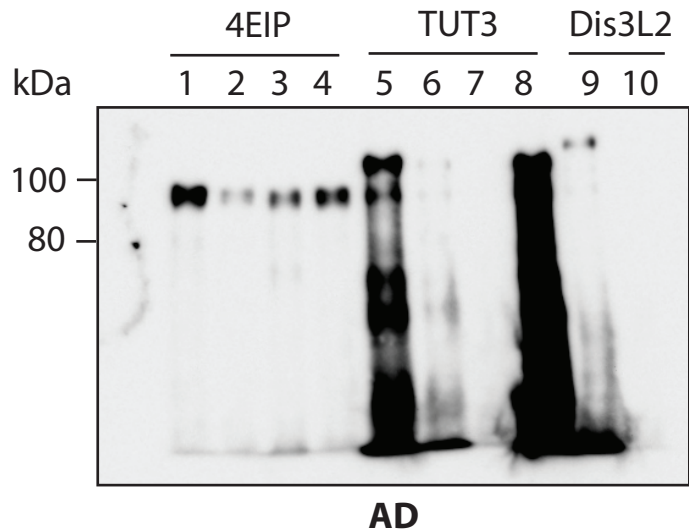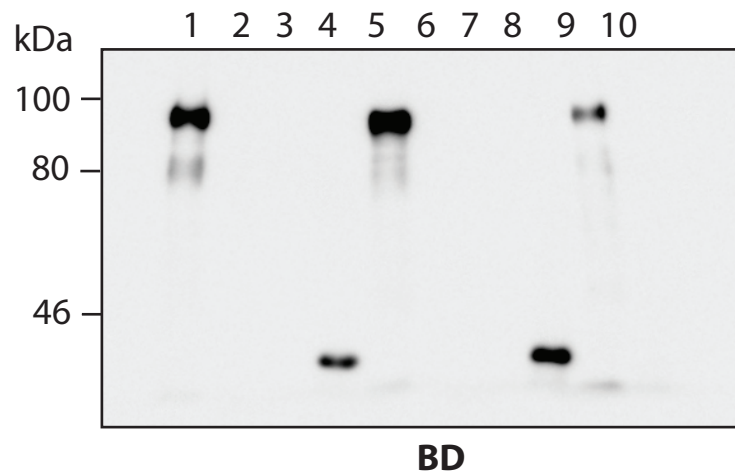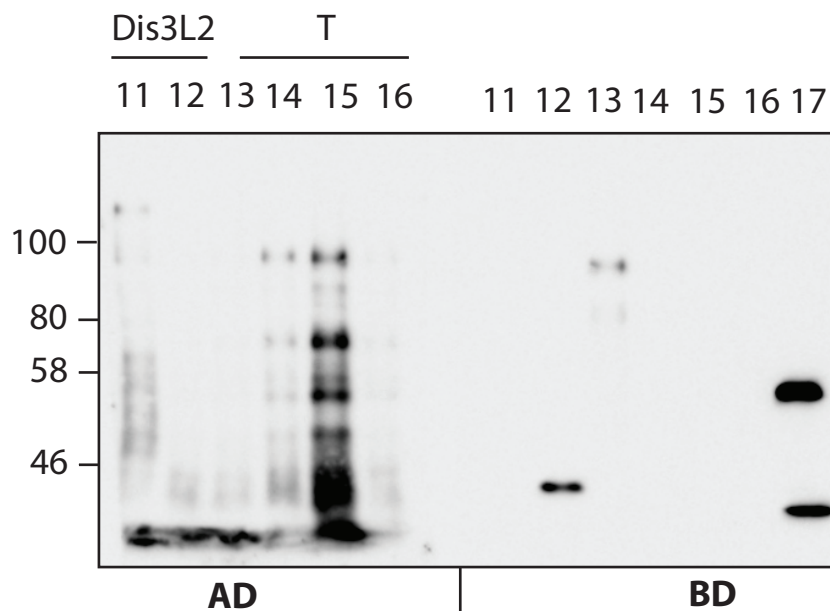

|              |        |
|--------------|--------|
| 1, 5, 9, 13  | 4EIP   |
| 2, 6, 10, 14 | TUT3   |
| 3, 7, 11, 15 | Dis3L2 |
| 4, 8, 12, 16 | LamC   |
| 17           | p53    |

Supplement: S3 Fig — Western blotting to show expression of fusion proteins in the yeast used in Fig 2E. (PDF) [file pone.0258903.s003.pdf]

# A

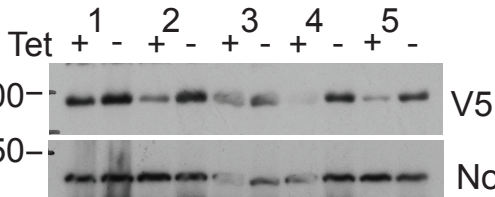

**B**

i 260 bp

ii 1094 bp

iii 555 bp

*TUT3*

*PUF3*

Control

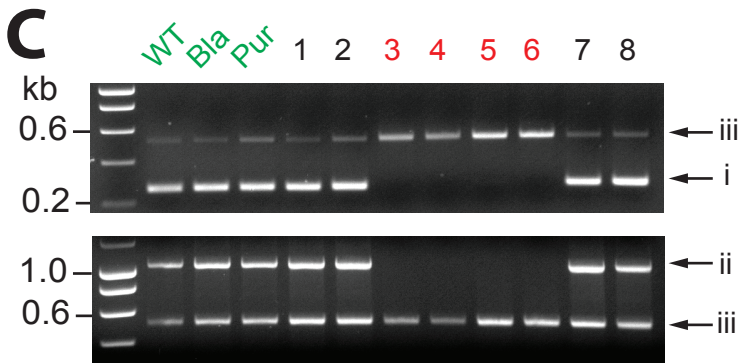

**D** TUT3<sup>-/-</sup> in Lister427 BSF

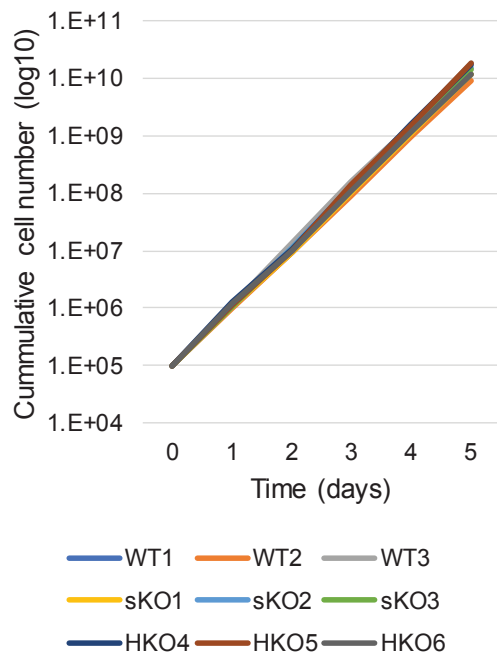

Supplement: S4 Fig — (A) Knockdown of TUT3 was induced in Lister 427 procyclic forms (PCFs) cultured in presence of tetracycline (+), with wild-type (WT) cells and RNAi cells cultured in absence of tetracycline serving as controls. The cell numbers were determined over the course of 5 days (upper panel), and knockdown efficiency was determined by western blotting (lower panel). (B) Primer pairs used for amplifying a 260 bp fragment within the TUT3 ORF (i) or a fragment spanning the 5’-UTR and the ORF (ii). Primers for amplification of the PUF3 gene were used as a control (iii). (C) Diagnostic polymerase chain reaction for the TUT3 gene. Each PCR contained primers to amplify PUF3 (purple, green, band iii) as a positive control. Product (i) was obtained from a primer pair (blue, cyan) that hybridised within the TUT3 open reading frame. Product (ii) was obtained using one primer that targets the ORF (orange) and another (red) that binds upstream of TUT3 to a sequence absent from the knockout plasmid. (D) The growth of monomorphic bloodstream forms with knockout of either a single copy or both copies of the TUT3 coding sequence (sKO and HKO, respectively) was monitored over the course of 5 days. (PDF) [file pone.0258903.s004.pdf]

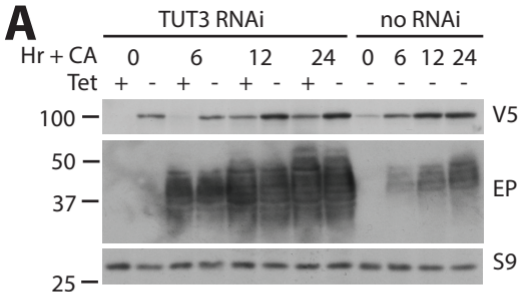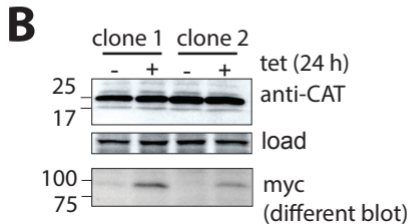

Supplement: S5 Fig — (A) Differentiation-competent T. brucei (EATRO1125 strain) with one V5-tagged TUT3 gene and RNAi targeting TUT3 were grown to a maximum density of 2 × 10^6^ cells/mL. 6 mM cis-aconitate (CA) was then added, and the temperature was reduced to 27°C to induce differentiation. Cultures were grown with or without tetracycline, with the drug being included for 24 h before addition of cis-aconitate. For each time point, 5 × 10^6^ cells were collected for western blot measurement of V5-TUT3, Ep procyclin, and a control rpotein, ribosomal protein S9. (B) Tethering of lambdaN-TUT3-myc has no effect on a box-B-containing reporter mRNA encoding chloramphenicaol acetyltransferase (CAT). Expression of lambdaN-TUT3-myc was induced for 24h then CATwas measured by Western blotting. A protein that cross-reacts with the anti-CAT antibody served as a loading control. (PDF) [file pone.0258903.s005.pdf]
