# Supplementary material for: The EIF4E1-4EIP cap-binding complex of Trypanosoma brucei interacts with the terminal uridylyl transferase TUT3
Source: PLoS One. 2021 Nov 22;16(11):e0258903. doi: 10.1371/journal.pone.0258903 (PMC8608314; doi:10.1371/journal.pone.0258903)
Supplement: S6 Fig — GPEET mRNA 3’-ends in normal and glucose-adapted WT procyclic forms (PCFs) were examined by cap removal, circularization, selective amplification, and DNA sequencing. Identified poly(A)-tailed GPEET mRNAs from low- and high-glucose-grown cells are shown; 20 sequences were analysed each. (PDF) [file pone.0258903.s006.pdf]

Supplementary file S6

Start of 5' UTR

Part of spliced leader

polyA tail

End of 3' UTR

20 analyzed each

Low glucose:

CGCCTCGGAGGAACGAAACCCCTTTGAAAAGGTGGCTTTCGTTTATATATTCTCCATCTGGTGCATCGTGTTTATTTCGTATTATTCCCTGTCAA
AAGCTGCGGATATTCATTTAATATTCTTTTCGTTATATATTTTTAGTTCTATCCTTTATTTTATCCGTTTTAATCGTCTTCTGAGACCCACAGCCCT
GTAGATTTCTGTGATGTTTCGGTTGCGTATTCCATAATTTTAANCGTTCCACTCCTATTTTTTTCATTCCTTTGAATTTGGATCTTNANNAAAATA
AAAAAAAAAAAAAAAAAAAAAAAAAAAAAAAAAGAAAAAAAAAAAAAAAAAAAAAAAAGTTANAACAGTTTCTGTACCATATNGACTTCAN
TTACNCCAAAAAGNAAAATTCNCAAGGGCNCCTCGTTCCCTTTNTCNGCNCGCTATTCTTCTGTTCAGCGCGAACNAAGGTCGTTAACNCGNG
GGGGGGCCCGGGGANAT

ATAGCGAGCAGATAAAGGGAACGAGGTGCCATTGTGAATTTTACTTTTTGGTGTAATTGAAGTCAATATAGTACAGAAACTGTTCTAATAATTT
TTTTTTTTTTTTTTTTTTTTTTTTTTTTTTTTTTTTTTTTTTTTTTTTTTTTTTTTTTTTTTTTTTTTTTTTTNAAA

GTGTAAAGCGCCTCGGAGGAACGAAACCCCTTTGAAAAGGTGGCTTTCGTTTATATATTCTCCATCTGGTGCATCGTGTTTATTTCGTATTATTC
CCTGTCAAAAGCTGCGGATATTCATTTAATATTCTTTTCGTTATATATTTTTAGTTCTATCCTTTATTTTATCCGTTTTAATCGTCTTCTGAGACCCA
CAGCCCTGTAGATTTCTGTGATGTTTCGGTTGCGTATTCCATAATTTTAAGCGTTTCACTTCTATTTTTTTTCATTCCTTTGAATTTGGATCTTAAA
AAAAAAAAAAAAAAAAAAAAAAAAAAAAAAAAAAAAAAAAAAAAAAAAAAAAAAAAAAAAAAAAAAAAAAAAAAAAAAAAAAAAANTT

CGGATGCAAGCGTGTAAAGCGCCTCGGAGGAACGAAACCCTTTGAAAAGGTTCCTTTCATTTATATCGCCTCCATATGGTGCATCGTGTTTGTT
TCCTGCTGTTTCTTGTAAAACAAGTGTGGACATTCATTTAATATTTTTTCGTTATATTTTTTTTGGTGACATCCTTTCTAATGCCTTATTGACCATC
GCCTGAGACCCACAGCCCTGTAGATTTCTGTGATGTTTCGGTTGCGTATTCCATAATTTTAANCGTTTCACTTCTATTTTTTTCATTCCTTTGAATT
TGGATCTTAAAAAAAAAAAAAAAAAAAAAAAAAAAAAAAAAAAAAAAAAAAAAAAAAAAAAAAAAAAAAAAAAAAAAAAAAAAAAAAAAAAA
AAAAAAAAAAAAAAAAAAAAAAAAAAAAAAAAA

TAGCGAGCAGATAAAGGGAACGAGGTGCCATTGTGAATTTTACTTTTTGGTGTAATTGAAGTCAATATAGTACAGAAACTGTTCTAATTTTTTT
TTTTTTTTTTTTTTTTTTTTTTTTTTTTTTTTTTTTTTTTTTTTTTTTTTTTTTTTTTTTTTTTTTTTTTTTTTTTTTTTTTTTTTTTTTTTTTTTTTTTTTTTTTTTTT
TTTTTTTTTTTTTTTN

GCGNGTAAAGCGCCTCGGAGGAACGAAACCCTTTGAAAAGGTTCCTTTCATTTATATCGCCTCCATATGGTGCATCGTGTTTGTTTCCTGCTGT
TTCTTGTAAAACAAGTGTGGACATTCATTTAATATTTTTTCGTTATATTTTTTTGGTGACATCCTTTCTAATGCCTTATTAACCATCGCCTGAGACC

CACAGCCCTGTAGATTTCTGTGATGTTTCGGTTGCGTATTCCATAATTTTAAGCGTTTCACTTCTATTTTTTTTCATTCCTTTGAATTTGGATCTTA
AAAAAAAAAAAAAAAAAAAAAAAAAAAAAAAAAAAAAAAAAAAAAAAAAAAAAAAAAAAAAAAAAAAAAANG

ATNNCGAGCAGATAAAGGGAACGAGGTGCCATTGTGAATTTTACTTTTTGGTGTAATTGAAGTCAATATAGTACAGAAACTGTTCTAATATTTT
TTTTTTTTTTTTTTTTTTTTTTTTTTTTTTTTTTTTTTTTTTTTTTTTTTTTTTTTTTTTTTTTTTTTTTTTTTTTTTTTTTTTTTTTTNNA

AAAGGGAACNAGGNGCCATTGTGAATTTTACTTTTTGGTGTAATTGAAGTCAATATAGTACAGAAACTGTTCTAATACTTTTTTTATTTTTTTTT
TTTTTTTTTTTTTTTTTTTTTTTTTTTTTTTTNCNTTTNCNTTTTTTTTTTTTTTTTTTTTTTTTT

High glucose:

GCGGATGCAAGCGTGTAAAGCGCCTCGGAGGAACGAAACCCTTTGAAAAGGTTCCTTTCATTTATATCGCCTCCATATGGTGCATCGTGTTTGT
TTCCTGCTGTTTCTTGTAAAACAAGTGTGGACATTCATTTAATATTTTTTCGTTATATTTTTTTGGTGACATCCTTTCTAATGCCTTATTAACCATC
GCCTGAGACCCACAGCCCTGTAGATTTCTGTGATGTTTCGGTTGCGTATTCCATAATTTTAAGCGTTTCACTTCTATTTTTTTTCATTCCTTTGAAT
TTGGATCTTAAAAAAAAAAAAAAAAAAAAAAAAAAAAAAAAAAAAAAAAAAAAAAAAAAAAAAAAAAAAAAAAAAAAAAAAAAAA
AAAAAAAAAAAAAAAAAAAAAAAAAAAAAAAAAAAAAAAAAAAAAAAAAAA

Note: Remaining amplicons were not GPEET
